# Effects of ozone nano-bubble water on mucositis induced by cancer chemotherapy

**DOI:** 10.1016/j.bbrep.2019.100697

**Published:** 2019-10-18

**Authors:** Kamichika Hayashi, Takeshi Onda, Hirona Honda, Natsuo Ozawa, Hitoshi Ohata, Nobuo Takano, Takahiko Shibahara

**Affiliations:** a Department of Oral and Maxillofacial Surgery, Tokyo Dental College, Japan; b Oral Cancer Center, Tokyo Dental College, Japan

**Keywords:** Cancer chemotherapy, Mucositis, Ozone nano-bubble water, Critical colonization, Bacterial microorganism

## Abstract

No effective, reliable treatment for stomatitis associated with cancer therapy has been established. This study focused on the its effectiveness of ozone nano-bubble water (ONBW) for the treatment of chemotherapy-induced stomatitis. Oral mucositis was induced in 14-week-old male Sprague-Dawley rats (N = 21). The animals were randomly divided into 3 groups: 7 without treatment (control); 7 treated with physiological salt solution (saline); and 7 treated with ONBW. Animals were weighed on Days 7, 9, 11, and 16. Stomatitis grade evaluation and bacterial count measurements were performed before rinsing in all animals 3, 5, and 10 days after acetic acid irritation (Days 9, 11, and 17 respectively). Weight loss after stomatitis creation was observed in all groups, with significant differences between the control and ONBW groups and between the saline and ONBW groups on Day 16. The stomatitis grade did not worsen during the experimental period in any group, with the lowest grades in the ONBW group on Days 11 and 16. Significant differences were identified between the control and ONBW groups and between the saline and ONBW groups on Days 11 and 16. Oral bacterial counts tended to decrease over time in all three groups, with the greatest decrease in the ONBW group, followed by the saline group. The decrease in the bacterial count was steepest in the ONBW group. Rinsing out the oral cavity with ONBW decreased bacterial counts and encouraged the healing of oral chemotherapy-induced stomatitis. ONBW may be an effective treatment for chemotherapy-induced stomatitis.

## Highlights

• An effective and reliable treatment for stomatitis associated with cancer chemotherapy has not been established so far.
• This study aimed to evaluate the effectiveness of ozone nano-bubble water (ONBW) for the treatment of chemotherapy-induced stomatitis.
• The efficacy of ONBW for treating chemotherapy-induced stomatitis was studied in a rat model.
• Rinsing out the oral cavity with ONBW decreased bacterial counts and encouraged the healing of oral chemotherapy-induced stomatitis.
• The present results suggest that ONBW may be an effective treatment for chemotherapy-induced stomatitis in the future.

## 1 Introduction

Cancer treatment has made great strides with the development of new anticancer agents and combination radiotherapy protocols [1]. Compared with the development of therapies for primary disease, however, the development of treatments for the side effects associated with these therapies is lagging [2]. It has been reported that oral mucositis is a common side effect in patients receiving anticancer agents, with an incidence of 25%–55% in patients receiving anticancer agents for solid cancer, 70%–90% in those receiving high-dose anticancer agents for hematopoietic stem cell transplantation, and almost 100% among patients receiving anticancer agents and radiotherapy of the head and neck [1,3,4]. The pain of stomatitis causes masticatory and swallowing problems that lead to poor nutrition, reducing patients’ quality of life (QOL) and making them more susceptible to infection [5]. Stomatitis is not only a dose-limiting factor [6], but it is also known to increase infection-related mortality [7], and it may necessitate the withdrawal of cancer treatment or changes to the treatment plan [8].

In general, the etiology of chemotherapy-induced stomatitis can be broadly divided into two main causes. One primary cause is mucosal inflammation due to the destruction of the cells that compose the oral mucosa by reactive oxygen species (superoxide or hydroxy radicals) generated by anticancer agents. Cells in the oral mucosa also undergo metabolic damage as a result of their uptake of anticancer agents, blocking healthy oral mucosal cell turnover. The secondary cause is the adhesion of high levels of oral bacteria to the ulcerated surface, causing local infection on the mucosal surface, and, in combination with the metabolic damage and susceptibility to infection caused by anticancer agents, this can become intractable or serious [9,10]. Stomatitis is being studied using a range of different animal models [11], but the detailed etiology of chemotherapy-induced stomatitis is unknown and may in fact be more complex [[12], [13], [14]].

As yet, no studies of stomatitis associated with cancer treatment with a high evidence level have been published, and an effective, reliable method of treatment has yet to be established [15,16]. There is a need for the development of a treatment method for chemotherapy-induced stomatitis to enable the completion of high-quality cancer treatment.

This study focused on ozone nano-bubble water (ONBW) [20,21], which has previously been shown to have a range of effects, including antibacterial effects [17], promotion of wound healing [18], an anti-inflammatory effect [18], and a hemostatic effect [19], and its effectiveness in the treatment of chemotherapy-induced stomatitis was investigated.

## 2 Materials and methods

### 2.1 Ozone nano-bubble water

Ozone nano-bubble water (REO Institute, Inc, Sendai, Japan) was generated using the production method described by Kamiyama [20]. Micro-bubbles with a diameter of less than 50 μm were generated in hard water (underground water) at a salinity of 0.9 mass%. The micro-bubbles were then rapidly crushed into bubbles with a diameter of less than 200 nm for use as the ONBW in this study.

### 2.2 Animals

All of the procedures performed with live animals conformed to the ethical guidelines established by the Japanese Council on Animal Care and were approved by the animal care committee of the Tokyo Dental College (Permit Number: 282403). Fourteen-week-old male Sprague-Dawley rats (N = 21) were obtained from Sankyo Laboratory (Tokyo, Japan). All animals were housed in a room maintained under standardized light (12:12 h light-dark cycle), temperature (23 ± 2 °C), and humidity (55% ± 5%) conditions with free access to food pellets and drinking water.

### 2.3 An animal model for mucositis induced by cancer chemotherapy

The protocol for the induction of oral mucositis was modified on the basis of a previously published protocol [22,23]. All rats received intraperitoneal administration of 5-FU (Wako Pure Chemical Industries Ltd, Osaka, Japan) (60 mg/kg/day) on Days 1, 2, 3, 4, and 5. On Day 6, after anesthesia induction with 4% sevoflurane (Maruishi Pharmaceutical Co., Ltd., Osaka, Japan) inhalation, the rats were further anesthetized by intraperitoneal injection with sodium pentobarbital (30 mg/kg body weight Somnopentyl; Kyoritsu Seiyaku, Tokyo, Japan), and 100% acetic acid (Wako Pure Chemical Industries Ltd, Osaka, Japan) 50 μl was painted onto the lingual dorsum with a Plaut brush® (Oral Care. Ltd, Tokyo, Japan) and rubbed in to produce stomatitis. The animals were randomly divided into 3 groups: 7 without treatment as the control animals, 7 treated with physiological salt solution (saline), and 7 treated with ONBW.

After stomatitis creation, the animals’ mouths were rinsed four times daily (every 6 h) with physiological saline in the saline group and with ONBW in the ONBW group, while the mouths of the control group were left untouched. Rinsing was carried out using a Doltz EW1211® (Panasonic Healthcare, Tokyo, Japan) at a water pressure of 4.0 kgf/cm^2^.

All animals were weighed on Days 7, 9, 11, and 16.

**Fig. 1:**
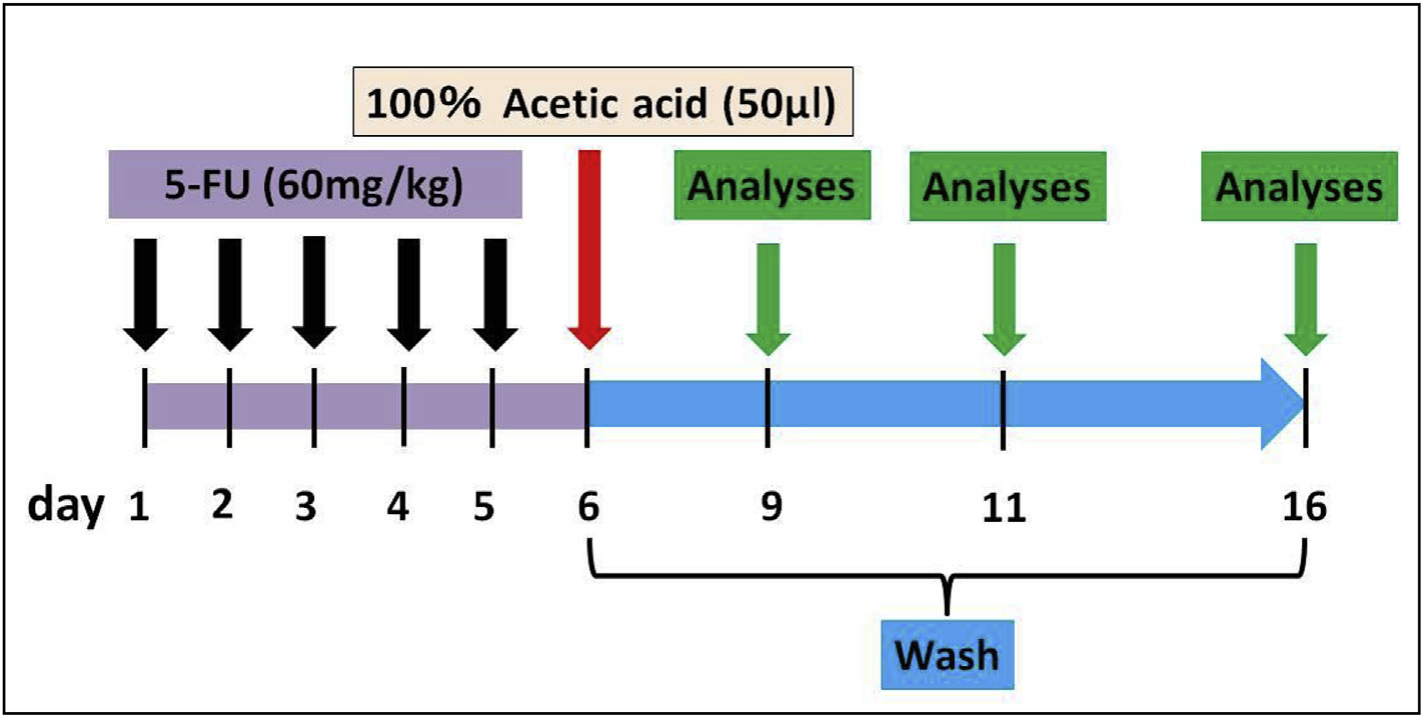
Experimental protocol. 5-FU (60 mg/kg/day) was administered on Days 1, 2, 3, 4, and 5. On Day 6, 100% acetic acid 50 μl was painted onto the lingual dorsum to irritate it and produce stomatitis. After stomatitis creation (Day 6), rinsing was carried out using a Doltz EW1211® (Panasonic Healthcare, Tokyo, Japan) at a water pressure of 4.0 kgf/cm^2^ four times daily until Day 16. Stomatitis grading and bacterial count measurement were carried out prior to rinsing 3, 5, and 10 days after acetic acid irritation (Days 9, 11, and 17 respectively).

Stomatitis grade evaluation and bacterial count measurements were also carried out before rinsing in all animals 3, 5, and 10 days after acetic acid irritation (Days 9, 11, and 17 respectively) (Fig. 1).

### 2.4 Stomatitis grading

**Fig. 2:**
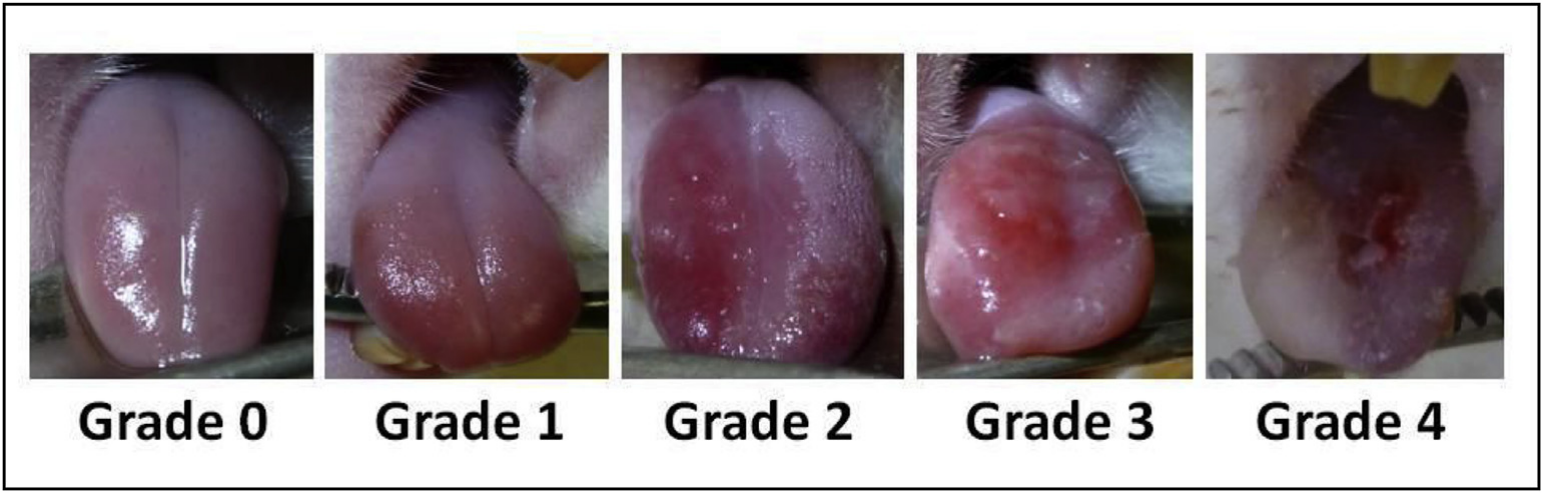
Rat chemotherapy-induced stomatitis grading criteria. Grade 0: Normal mucosa Grade 1: Redness of the mucosa with punctate ulcers or pseudomembrane Grade 2: Confluent ulceration or pseudomembrane, no bleeding on slight stimulation Grade 3: Confluent ulceration or pseudomembrane, bleeding on slight stimulation. Grade 4: Tissue necrosis or spontaneous bleeding.

The National Cancer Institute-Common Terminology Criteria for Adverse Events (NCI-CTCAE) evaluation criteria for oral mucositis [24] were modified to produce criteria for grading chemotherapy-induced oral mucositis in rats for this study (Fig. 2). Stomatitis grading using these criteria was carried out before rinsing 3, 5, and 10 days after acetic acid irritation (Days 9, 11, and 17 respectively).

### 2.5 Criteria for oral mucositis induced by cancer chemotherapy in rats

Grade 0: Normal mucosa.
Grade 1: Redness of the mucosa with punctate ulcers or pseudomembrane.
Grade 2: Confluent ulceration or pseudomembrane, no bleeding on slight stimulation.
Grade 3: Confluent ulceration or pseudomembrane, bleeding on slight stimulation.
Grade 4: Tissue necrosis or spontaneous bleeding.

### 2.6 Oral bacterial count measurement

Oral bacterial count measurement was carried out prior to rinsing 3, 5, and 10 days after acetic acid irritation (Days 9, 11, and 17 respectively). Bacteria in the oral cavity were measured in a standardized way based on a previous study. The amount of bacteria on the following places in the oral cavity was measured by a bacteria detection apparatus [25] (Panasonic Healthcare, Tokyo, Japan). Briefly, a sterilized swab was pressed on the sampling area and rubbed backwards and forwards three times along the center of the lingual dorsum from the lingual apex to the lingual root with a constant force of 20 g using a device on the bacteria detection apparatus. The swab was swiped on the applicable areas three times in a 10-mm swath. The swab was then placed in distilled water in the bacteria detection apparatus for counting. Bacteria quantification was performed using the dielectrophoretic impedance measurement technique [26,27].

### 2.7 Statistical analyses

The distributions of weight (g) on Day 6 and bacterial count ( × 10^5^ cfu/ml) on Day 9 were tested for normality using the Shapiro-Wilk test. If the normality test showed a significant difference, non-parametric methods were used, and if no significant difference was found, parametric methods were applied.

Basic statistics for weight and stomatitis grading were calculated for each group, and differences between groups and time points were compared using non-parametric methods. Differences among all three groups were investigated using the Kruskal-Wallis test, and comparisons between two groups were made using the Mann-Whitney *U* test, with *p* values adjusted using a non-parametric version of Tukey's method (Steel-Dwass test).

Basic statistics for oral bacterial counts were calculated for each group, and differences between groups and time points were compared using parametric methods. Differences among all three groups were investigated using analysis of variance (ANOVA), and comparisons between two groups were made using the *t*-test, with *p* values adjusted using Tukey's method.

SAS 9.4 statistical software (SAS Institute Inc., Cary, NC, USA) was used for statistical analysis, with p < 0.05 (two-tailed) regarded as significant.

## 3 Results

### 3.1 Data distributions

The Shapiro-Wilk tests for normality of the distributions of weight (g) on Day 6 and bacterial count ( × 10^5^ cfu/ml) on Day 9 showed a significant difference for weight, but not for bacterial count.

Weight: p = 0.0052.

Bacterial count: p = 0.7728.

### 3.2 Evaluation of weight changes

Weight loss after stomatitis creation was observed in all groups. Weight was lowest on Day 9, and no great change was seen on Day 11, but by Day 16 it had started to increase again.

Basic statistics for weight were calculated for each group, and differences between groups and time points were compared using non-parametric methods. Differences among all three groups were investigated using the Kruskal-Wallis test, and comparisons between two groups were made using the Mann-Whitney *U* test, with *p* values adjusted using a non-parametric version of Tukey's method (Steel-Dwass test).

Significant differences were identified between the control and ONBW groups and between the saline and OBNW groups on Day 16 (*p* < 0.05).

### 3.3 Stomatitis grading

The stomatitis grade did not worsen during the experimental period in any of the groups. The lowest grades were seen in the OBNW group on Days 11 and 16. In the control group, there was no improvement in grade during the experimental period. In the saline group, there was no great change on Day 11, but the grade improved on Day 16. In the ONBW group, the grade improved on both Day 11 and Day 16, with an overall improvement in grade over time.

**Fig. 3:**
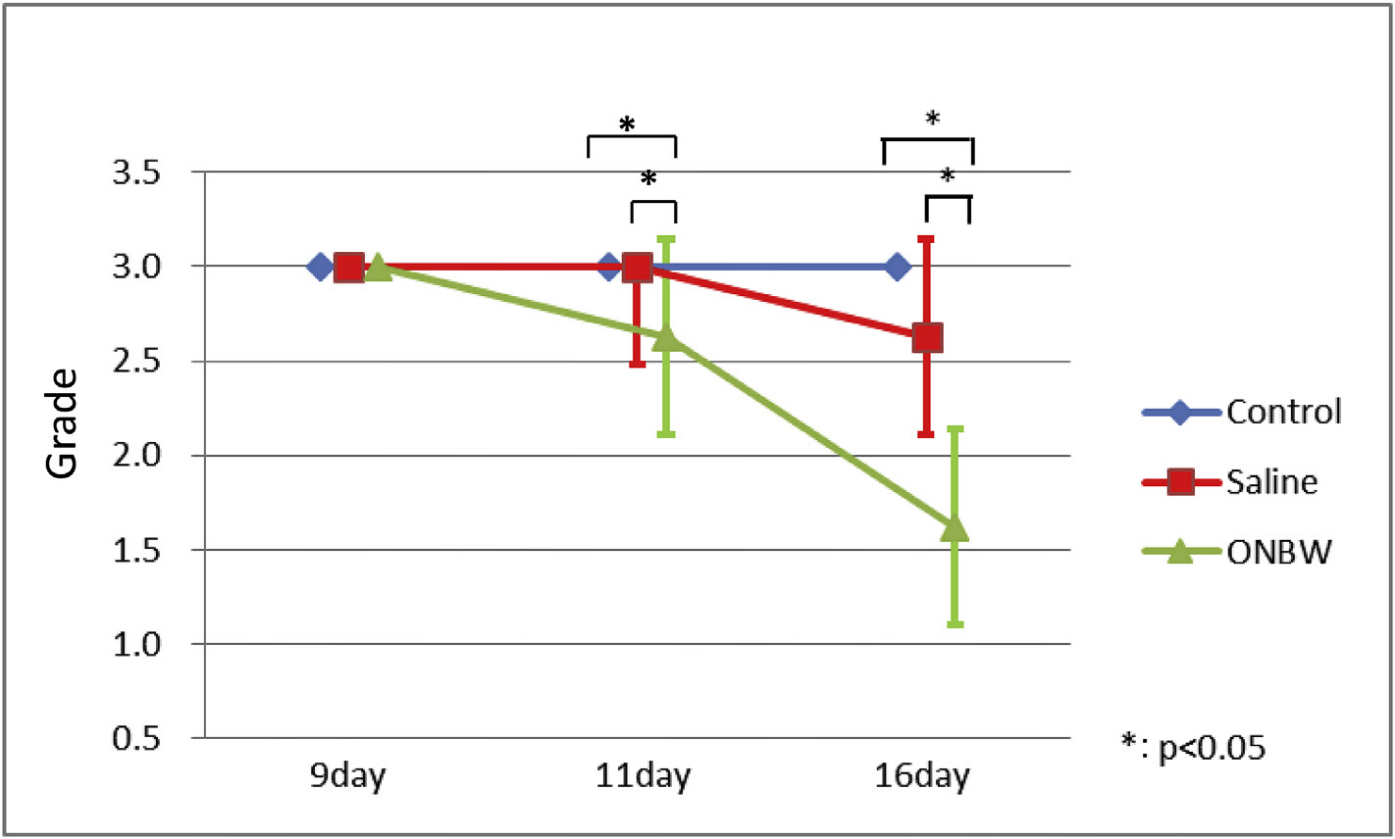
Stomatitis grading. The stomatitis grade did not worsen during the experimental period in any of the groups. The lowest grades are seen in the OBNW group on Days 11 and 16. In the control group, there is no improvement in grade during the experimental period. In the saline group, there is no great change on Day 11, but the grade improves on Day 16. In the ONBW group, the grade improves on both Day 11 and Day 16, with an overall improvement in grade over time. Basic statistics for stomatitis grading were calculated for each group, and differences between groups and time points were compared using non-parametric methods. Differences between all three groups were investigated using the Kruskal-Wallis test, and comparisons between two groups were made using the Mann-Whitney *U* test, with *p* values adjusted using a non-parametric version of Tukey's method (Steel-Dwass test). Significant differences were identified between the control and ONBW groups and between the saline and OBNW groups on Days 11 and 16 (*p* < 0.5).*: p < 0.05.

Significant differences were identified between the control and ONBW groups and between the saline and OBNW groups on Days 11 and 16 (Fig. 3).

### 3.4 Oral bacterial count measurements

Oral bacterial counts tended to decline over time in all three groups. The greatest decrease was seen in the ONBW group, followed by the saline group. The decline in the bacterial count was steepest in the ONBW group.

**Fig. 4:**
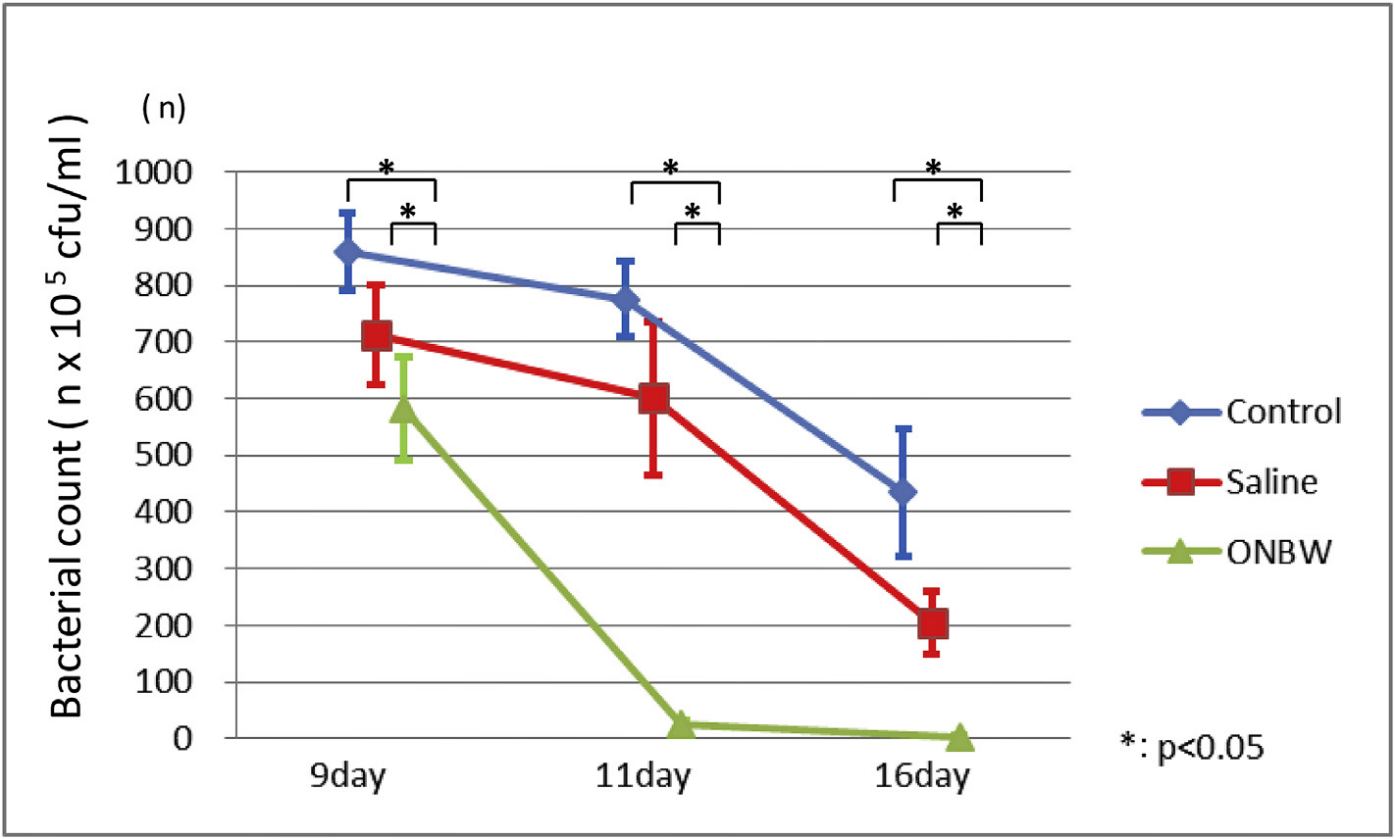
Oral bacterial count measurements. The oral bacterial count tends to decline over time in all three groups. The greatest decrease is seen in the ONBW group, followed by the saline group. The steepest decline in bacterial count is seen in the ONBW group. Basic statistics for oral bacterial count were calculated for each group, and differences between groups and time points were compared using parametric methods. Differences among all three groups were investigated using analysis of variance (ANOVA), and comparisons between two groups were made using the *t*-test, with *p* values adjusted using Tukey's method. Significant differences are seen between the control and OBNW groups and between the saline and OBNW groups on Days 9, 11, and 16 (*p* < 0.5).*: p < 0.05.

Significant differences were identified between the control and OBNW groups and between the saline and OBNW groups on Days 9, 11, and 16 (Fig. 4).

## 4 Discussion

The cortical layer of the oral mucosa is covered with stratified squamous epithelium, below which lies fibrous connective tissue containing a large number of capillary vessels. Wound healing in the oral mucosa follows a process of vasoconstriction, blood clot formation, fibrin formation, inflammatory cell infiltration, cell proliferation, neovascularization, and epithelial regeneration. Local factors affecting this healing process include insufficient oxygen supply, local infection, and the presence of foreign bodies. Systemic factors that may also have an effect include age, sex, circulatory impairment, immunocompromised status, nutritional status, systemic disease, and use of concomitant medications such as steroids and anticancer agents [28]. Sonis [10] found that local mucosal infection by oral resident bacteria is an aggravating factor in chemotherapy-induced stomatitis, and the importance of oral care has become a focus in ensuring that cancer patients can complete good-quality therapy. One characteristic of chemotherapy-induced stomatitis is the adhesion of high levels of oral bacteria to the ulcerated surface, causing local infection of the mucosal surface, where the bacteria become established and flourish. In combination with the metabolic damage and susceptibility to infection caused by anticancer agents, this can become intractable or serious [9,10]. Local infection thus delays the healing of stomatitis, and the delayed healing of stomatitis also increases susceptibility to infection. In chemotherapy-induced stomatitis, local infection and delayed healing of stomatitis act synergistically to bring about critical colonization [29]. Reducing the bacterial count is important to improve critical colonization, and this can be achieved by chemical removal with pharmaceutical agents or physical removal by rinsing or similar methods. Although animal models of chemotherapy-induced stomatitis have previously been reported [11], no previous study has addressed the association between the healing of chemotherapy-induced stomatitis and bacterial count.

There are two reasons that critical colonization occurs in chemotherapy-induced stomatitis. The first is the presence of large numbers of resident bacteria in the mouth, in addition to the fact that the oral mucosa is constantly covered with mucus. The second is that, in stomatitis, the ulcerated surfaces are covered with necrotic material, which offers a breeding ground for bacteria and creates an environment in which they can easily become established. The fact that the ulcerated surface is covered in mucus and necrotic material is believed to weaken the effect of pharmaceutical agents [[30], [31], [32], [33]]. In the present study, the bacterial counts in both the saline and OBNW groups decreased compared with the control group. This may have been because of the removal of mucosal substances and necrotic material by the water pressure generated during rinsing with the Doltz EW1211, which exerted a mechanical cleaning effect [34].

Previous studies have described the excellent antibacterial effect of ONBW [7,21,35]. Unlike antibiotics, ONBW has the advantage that it does not risk the potential emergence of drug-resistant bacteria [36]. In the present study, the bacterial count decreased markedly in the ONBW group, suggesting that it may have exerted an antibacterial effect on the bacteria on the mucosal surface in the chemotherapy-induced stomatitis. However, in the present study, which bacterial strains were present in chemotherapy-induced stomatitis, or which strains were affected to what extent by the antibacterial effect of ONBW were not investigated, and further investigation of these questions is required.

There was no significant difference among the three groups in the weight changes on Days 9 and 11, and no difference in the amount eaten was observed. After stomatitis developed, the amount eaten by the animals decreased, causing them to lose weight. This may have been because of the pain during eating associated with stomatitis. On Day 16, the weight of the animals in the ONBW group increased significantly compared with the control and saline groups, which may have been because they were eating more as the stomatitis had healed, meaning that they were suffering less pain. It was considered unlikely that ONBW had had an analgesic effect on the pain during eating caused by stomatitis.

There was no increase in stomatitis grade during the experimental period, and the grade tended to decrease over time. No side effects or other adverse events occurred in the ONBW group during the experimental period, suggesting that ONBW does not cause any biological damage, but more in-depth *in vivo* studies at the cellular molecular level are required.

The MASCC/ISOO Evidence Based Clinical Practice Guidelines for Mucositis Secondary to Cancer Therapy suggest oral care, oral cryotherapy, low-level laser therapy, benzydamine mouthwash, and the use of recombinant human Keratinocyte Growth Factor-1 (KGF-1) as supportive therapies for preventing stomatitis associated with cancer treatment [37]. As yet, however, no studies of stomatitis associated with cancer treatment with a high evidence level have been published, and an effective, reliable, and biologically safe method of treatment has yet to be established [15,16,37]. The results of the present study suggest that ONBW may exert a strong antibacterial effect while causing no biological damage, promoting the healing of chemotherapy-induced stomatitis. Although more detailed investigations are required of the bacterial strains targeted by ONBW, a potential harmful effect at the cellular level, and its use in conjunction with a variety of different anticancer agents, the present results suggest that ONBW may be an effective treatment for chemotherapy-induced stomatitis.

## 5 Conclusions

The efficacy of ONBW for treating chemotherapy-induced stomatitis was investigated in a rat model. ONBW caused no biological harm and showed a strong antibacterial effect.

Rinsing out the oral cavity with ONBW decreased bacterial counts and encouraged the healing of oral chemotherapy-induced stomatitis.

The present results suggest that ONBW may be an effective treatment for chemotherapy-induced stomatitis in the future.

## Funding

This research did not receive any specific grant from funding agencies in the public, commercial, or not-for-profit sectors.

## Declaration of competing interest

The authors declare no conflicts of interest associated with this manuscript.
